# Dissecting cytosine methylation mechanics of dysmetabolism

**DOI:** 10.18632/aging.101788

**Published:** 2019-01-23

**Authors:** Chiara Cencioni, Carlo Gaetano, Francesco Spallotta

**Affiliations:** 1 National Research Council, Institute of Cell Biology and Neurobiology, Monterotondo, Rome, Italy; 2 Laboratorio di Epigenetica, Istituti Clinici Scientifici Maugeri, Pavia, Italy; 3 Cancer Epigenetics Laboratory, Candiolo Cancer Institute-FPO, Candiolo, Turin, Italy

**Keywords:** DNA methylation, hyperglycemia, epigenetics, aging, cancer

DNA might be covalently modified by epigenetic enzymes-dependent conversion of cytosine to 5-methylcytosine (5mC) [1]. Iteratively, 5mC might be oxidized to 5-hydroxylmethylcytosine (5hmC), 5-formylcytosine (5fC) and 5-carboxylcytosine (5caC). The final restoration of unmodified cytosines is triggered by thymine DNA glycosylase (TDG)-dependent activation of the base excision repair pathway [1]. The cytosine methylation/demethylation cycle plays a pivotal role during several cellular processes, including maintenance of genome stability, differentiation, X chromosome inactivation and the functional regulation of specific genomic regions/loci during cell proliferation and lifespan [1,2]. Although dynamically introduced into the genome and tightly regulated, modified cytosines might become stable DNA derivatives directly affecting chromatin structure and epigenetic landscape [2]. Specifically, *de novo* DNA methyltransferases DNMT3A and DNMT3B establish the methylation pattern during early development, maintained by DNMT1 during somatic cell division stage [3]. DNMT activity depends on availability of the universal methyl donor S-adenosyl-L-methionine (SAM), a metabolite synthesized by methionine pathway [3]. Conversely, DNA demethylation occurs either passively by methylation maintenance failure, or actively by iterative oxidation mediated by Ten-eleven-translocation (TET) enzymes 1, 2 and 3 (TET1, TET2 and TET3) [1,3]. TET isoforms have distinct and overlapping developmentally-related and organ-specific functions where their enzymatic activity is carried out by using ferrous ion (Fe^2+^) and α-ketoglutarate (αKG), a key metabolite of Krebs cycle [1,3]. Metabolism substantially harnesses the DNA (de)methylation cycle. Indeed, pathophysiological conditions associated with dysmetabolism (including metabolic syndrome, diabetes, cancer and other aging-associated chronic diseases) show tissue specific DNA (de)methylation alterations. In this regard, we recently reported about a reduction of intracellular αKG synthesis in *ex vivo* cultured cardiac mesenchymal cells (CMSCs) derived from type 2 diabetic donors. This αKG reduction is responsible for TET functional deregulation in response to hyperglycemia, TET1/TDG complex disassembly and the consequent decrease in TDG activity. Intriguingly, in our experimental condition, not only TET function depended on αKG availability, but we reported the unprecedented observation that αKG also acted as an allosteric TDG activator. Although originally observed *ex vivo*, these molecular alterations were validated in animal models of diabetes, hyperglycemia or metabolic syndrome providing additional ground to the so-called “hyperglycemic memory”, where iteratively oxidized forms of methylated cytosines might stably accumulate in the cardiac stroma. In fact, diabetic CMSCs and the heart of hyperglycemic mice showed higher levels of oxidized cytosines, evaluated both globally and at CpG island level in cell cycle and metabolically relevant gene promoters. In this condition, supplementing a cell-permeable form of αKG or administering intraperitoneally the (S)-2-[(2,6-dichlorobenzoyl) amino succinic acid (AA6), an unprecedented inhibitor of the αKG dehydrogenase, the altered methylation phenotype was rescued both *in vitro* and *in vivo*. The reactivation of the TET1/TDG complex triggered DNA demethylation improving glucose uptake, insulin response, and cell function [4]. These observations were, at least in part, confirmed in another study in which the reliance of DNA (de)methylation cycle on metabolism emerged clearly after diabetic patient stratification according to glycemic control. Indeed, well-controlled patients showed no difference in 5mC and 5hmC levels compared to healthy donors, whereas poorly-controlled patients accumulated more 5mC and 5hmC in peripheral blood mononucleated cells (PBMCs) independently from age, sex, lifestyle and years from diabetes initial diagnosis [5].

Intracellular αKG levels and TET function are influenced by glucose availability and uptake. Reduction in TET2 protein has been observed upon hyperglycemia as possibly consequence of AMPK kinase inactivation. In this study [6], AMPK phosphorylates TET2 at serine residue 99 protecting the enzyme from calpain-dependent degradation. Dissimilar from prior observations including ours [4,5], in PBMCs of diabetic patients, TET2 destabilization led to 5hmC level reduction, whereas 5mC levels did not changed. Here, the deregulation of DNA demethylation cycle affected the expression of cell cycle genes, oncosuppressors and oncogenes, providing evidences of a link between hyperglycemia and cancer predisposition. Indeed, TET2 seems working as tumor suppressor maintaining the 5mC/5hmC balance, whose alteration represents an important hallmark of cancer. In this light, this work provided the first evidence that metformin might exert anticancer activities preventing tumor growth by regulation of the AMPK-TET2-5hmC axis. Interestingly, aberrant DNA methylation profiles associated with cancer might derive not only from the deregulation of DNA (de)methylation enzymatic machinery, but also from an alteration of their cooperation. Hence, the cyclic distribution of the cytosine derivatives might be cooperatively determined by the integrated activities of DNA methylation-related enzymes. For this reason, their uncoordinated expression might represent another epigenetic hallmark of cancer [7]. In this regard, the chronological alteration of DNA methylation pattern is a well-recognized hallmark of aging. Indeed, the function of the DNA demethylation machinery declines in the elderly as consequence of reduction in TET1, TET3 and TDG gene expression. This decline does not depend on promoter methylation mechanisms rather on post-translational modifications or changes in the availabilities of crucial metabolites including αKG and SAM. Aging, in fact, is commonly associated with decreased αKG levels leading to 5hmC decrease and 5caC accumulation. These events might contribute to the exploitation of aberrant epigenetic and transcriptional programs and to the repression of the immune cell functions typical of elderly people [8].

**Figure 1:**
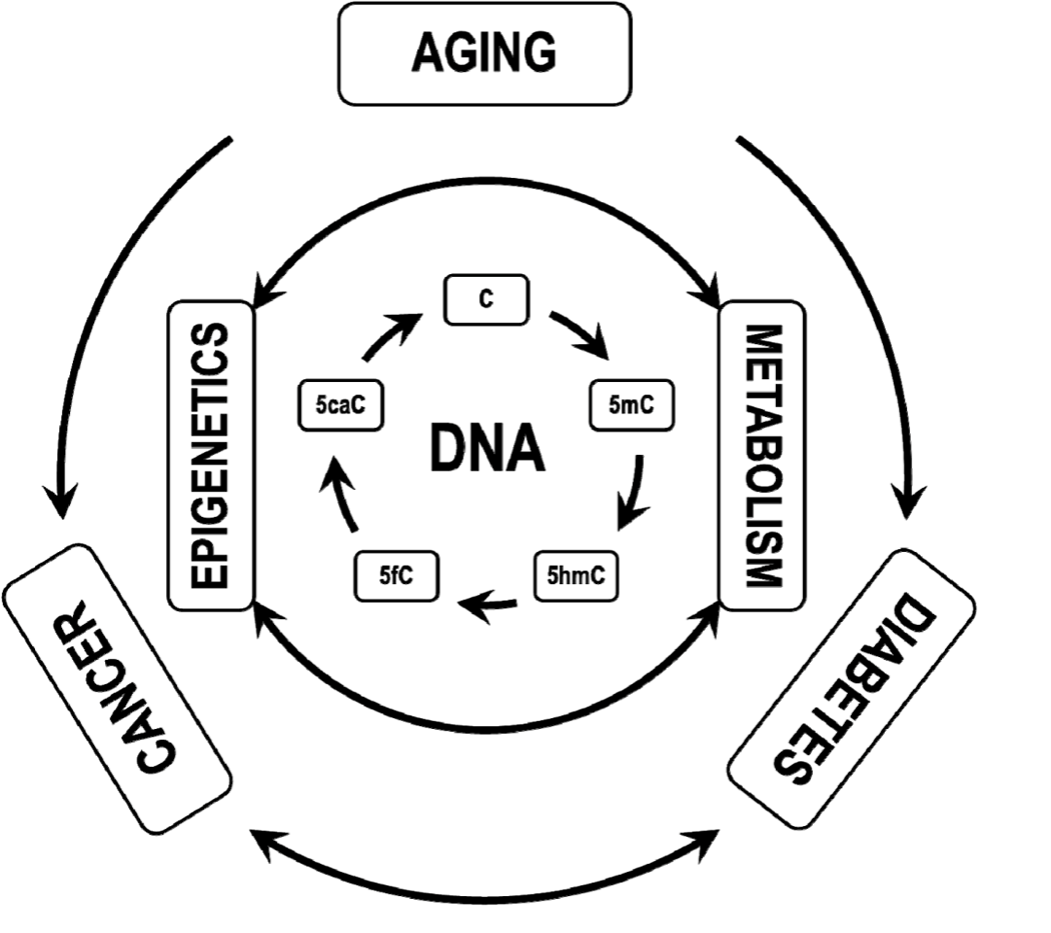
**The DNA (de)methylation cycle is finely regulated by the tight interconnection between epigenetics and cellular metabolism.** Its derangement alters cell transcriptome, leading to or worsening dysmetabolic conditions, including diabetes and cancer.

These experimental evidences, all arising from the tight interconnection between epigenetics and metabolism, point out the necessity of more studies investigating the DNA (de)methylation cycle under metabolically altered conditions. A metabolic derangement, in fact, influences the epigenetic enzyme machinery introducing unscheduled changes in the epigenome with consequences for the transcriptome possibly at the base of diabetes, cancer and aging-associated diseases (Figure 1).
