# Correction: Dissecting cytosine methylation mechanics of dysmetabolism

**DOI:** 10.18632/aging.101950

**Published:** 2019-04-30

**Authors:** Chiara Cencioni, Carlo Gaetano, Francesco Spallotta

**Affiliations:** 1 National Research Council, Institute of Cell Biology and Neurobiology, Monterotondo, Rome, Italy; 2 Laboratorio di Epigenetica, Istituti Clinici Scientifici Maugeri, Pavia, Italy; 3 Cancer Epigenetics Laboratory, Candiolo Cancer Institute-FPO, Candiolo, Turin, Italy

Original article: Aging (Albany NY) 2018; 11: 837-838

**This article has been corrected:** The corresponding author, Francesco Spallotta, requested to uniform his affiliation according to the request of the Italian Ministry of Health. The updated affiliation is provided below. The authors declare that this correction does not change the results or conclusions of this paper. The authors sincerely apologize for this error.

^3^Cancer Epigenetics Laboratory, Candiolo Cancer Institute, FPO - IRCCS - Candiolo, Turin 10060, Italy

